# *EPAS1* Variations and Hematological Adaptations to High-Altitude Hypoxia in Indigenous Goats in Yunnan Province, China

**DOI:** 10.3390/ani15050695

**Published:** 2025-02-27

**Authors:** Li Zhu, Lin Tang, Yunong Zhao, Shanshan Li, Xiao Gou, Weidong Deng, Xiaoyan Kong

**Affiliations:** 1 Faculty of Animal Science and Technology, Yunnan Agricultural University, Kunming 650201, China; zhuli18328815855@163.com (L.Z.); zero--xc@163.com (L.T.); 2 School of Animal Science and Technology, Foshan University, Foshan 528231, China; lightrainooo@163.com (Y.Z.); 15036126218@163.com (S.L.); gouxiaosa@163.com (X.G.)

**Keywords:** Tibetan goat, *EPAS1* gene, genetic polymorphism, hematological traits, hypoxia adaptation

## Abstract

The *EPAS1* gene plays a central role in hypoxia adaptation in high-altitude animals. Using over 400 blood samples from goats across elevations in Yunnan (500–3500 m), this study examined hematological traits, genetic polymorphisms, and protein structure. Red blood cell (RBC) and hemoglobin (HGB) levels increased significantly with altitude (*p* < 0.05), reflecting improved oxygen transport. A non-synonymous SNP (*g.86650 A>T*, p.Gln556Leu) exhibited adaptive selection, with the *T* allele frequency rising at higher altitudes (*p* < 0.05). At 2500 m, *TT* genotype goats showed significantly higher RBC and HGB levels than AA genotypes (*p* < 0.05). Protein modeling revealed structural instability caused by the polymorphism, highlighting its role in enhancing hypoxia adaptation. These findings provide a foundation for improving high-altitude livestock genetics.

## Simple Summary

Tibetan goats, native to high-altitude regions, have developed unique physiological and genetic adaptations to survive under low oxygen conditions. This study investigated the Endothelial PAS Domain Protein1 (*EPAS1*) gene, a critical regulator of hypoxia response, using blood samples from goats across elevations in Yunnan (500–3500 m). A key polymorphism (*g.86650 A>T*, p.Gln556Leu) was identified, with its frequency increasing at higher altitudes. This polymorphism was associated with enhanced oxygen transport (e.g., increased red blood cell count and hemoglobin concentration) while maintaining blood flow efficiency by reducing erythrocyte aggregation. Structural modeling confirmed its functional significance in hypoxia adaptation, offering valuable insights into genetic mechanisms underlying high-altitude survival.

## 1. Introduction

High altitudes exceeding 2500 m are defined as plateau regions, representing the critical elevation at which arterial oxygen saturation (SaO_2_) declines significantly [1]. These regions are characterized by extreme environmental factors, including low temperature, low humidity, intense ultraviolet radiation, low atmospheric pressure, and hypoxia, which present formidable challenges to the survival and reproduction of flora and fauna [2,3,4]. Among these factors, hypoxia is regarded as the primary environmental stressor, directly limiting physiological processes related to oxygen transport and utilization. For instance, at an altitude of 3700 m, oxygen partial pressure is only 63% of that at sea level [5], often leading to hypoxemia [6]. Investigating the mechanisms underlying animal adaptation to such extreme environments not only unveils the principles of molecular evolution but also provides essential theoretical guidance for the development of plateau animal husbandry.

In recent years, studies on plateau adaptability have identified multiple candidate genes, with *EPAS1*, Egl Nine Homolog 1 (*EGLN1*), and Protein Kinase AMP-Activated Catalytic Subunit Alpha 1 (*PRKAA1*) playing pivotal roles in the hypoxia-inducible factor (HIF) pathway [7,8,9,10,11,12,13,14]. The HIF pathway serves as the central regulatory mechanism in hypoxic environments, mediating erythropoiesis, angiogenesis, and metabolic processes to help organisms maintain oxygen homeostasis [15,16]. In both high-altitude human populations [10,17,18,19,20] and animals (e.g., yak, Tibetan sheep, and Tibetan goats) [11,21,22,23,24], the *EPAS1* gene has been confirmed as a key gene exhibiting strong signals of selection for plateau adaptation. *EPAS1* encodes HIF-2α, which regulates hemoglobin synthesis (HGB), oxygen transport, and energy metabolism, playing a vital role in high-altitude adaptation [25,26]. Non-synonymous single nucleotide polymorphisms (SNPs), such as H194R, have been significantly associated with relatively lower hematocrit in Andean highlanders [19].

Tibetan goats, traditional livestock in the Tibetan Plateau [27], inhabit regions above 4000 m, displaying enhanced cardiopulmonary function, oxygen-carrying capacity, and metabolic efficiency [28]. For example, Song et al. [29] demonstrated that the Q579L variation of the *EPAS1* gene significantly influences mean corpuscular hemoglobin concentration (MCHC) in Tibetan cashmere goats. In Nepalese goats, the frequency of the T allele at the Q579L variation increases significantly with altitude [30], suggesting a strong association between this variation and high-altitude adaptation.

The aim of this study was to detect and analyze *EPAS1* gene polymorphism in Yunnan goats distributed along a continuous altitudinal gradient and to explore its potential association with blood physiological parameters, including hematocrit level, hemoglobin concentration, and oxygen saturation. Moreover, protein structure prediction was conducted, focusing on the potential impact of the analyzed variant on the EPAS1 protein. These comprehensive analyses provide new theoretical insights into the mechanisms of high-altitude adaptation in Yunnan goats and offer valuable scientific support for the genetic improvement and sustainable development of plateau animal husbandry.

## 2. Materials and Methods

### 2.1. Selection of Experimental Animals and Measurement of Hematological Parameters

**Figure 1:**
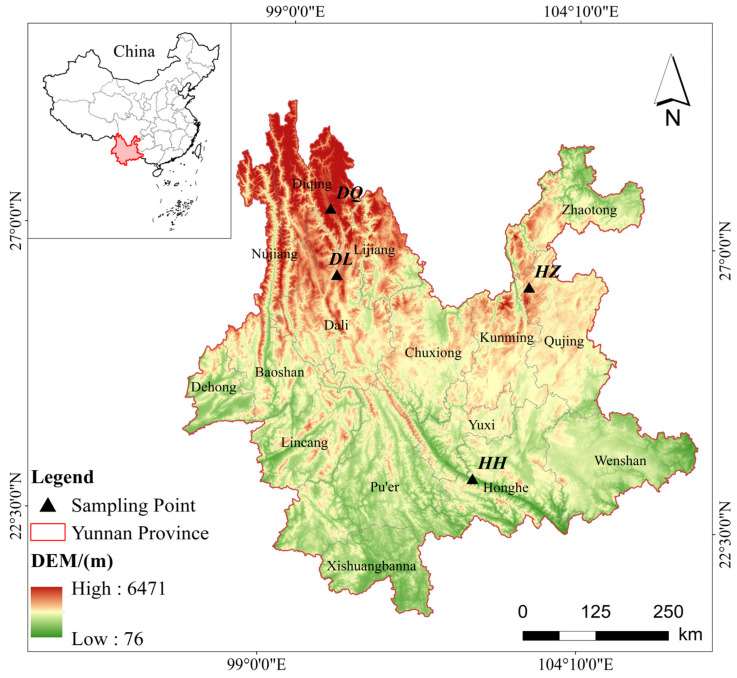
Geographic distribution and sample information of local goats at different altitudes in Yunnan Province.

A total of 407 blood samples from local indigenous Yunnan goats (healthy and adult) distributed along a continuous altitudinal gradient were collected for this study. All blood samples were collected from the jugular vein following standard veterinary procedures. This study adhered to animal welfare guidelines and was approved by the relevant ethical committee, ensuring that all animal handling and sample collection followed the principles of humane treatment and minimal distress. The samples were obtained from Yunnan Province, covering Honghe County (HH, 500 m, n = 61), Huize County (HZ, 1500 m, n = 109), Dali Prefecture (DL, 2500 m, n = 125), and Diqing Prefecture (DQ, 3800 m, n = 112). Detailed sample information is provided in Figure 1. Blood physiological parameters were measured using an automated veterinary hematology analyzer (Mindray, BC-30Vet, Shenzhen, China), which included red blood cell (RBC), hemoglobin concentration (HGB), hematocrit level (HCT), mean corpuscular volume (MCV), mean corpuscular hemoglobin concentration (MCHC), mean corpuscular hemoglobin (MCH), and red cell distribution width coefficient of variation (RDW). A total of seven parameters were assessed. Additionally, blood and plasma viscosity parameters were measured using a fully automated blood rheometer (Zhongchi, ZL9000, Beijing, China), combined with a cone-plate whole-blood testing system and a capillary plasma testing system. These included fibrinogen (FB), plasma viscosity (PV), whole-blood low-shear relative index (WLS), whole-blood medium-shear relative index (WMS), whole-blood high-shear relative index (WHS), erythrocyte aggregation index (EI), erythrocyte aggregation coefficient (EC), Casson viscosity (CV), red blood cell intraviscosity (RBCIV), low-shear flow resistance (LSFR), medium-shear flow resistance (MSFR), high-shear flow resistance (HSFR), and yield stress (YS), covering a total of 13 parameters.

To identify polymorphic variation in the goat genome, DNA was extracted from 10 individuals of both local Honghe goats (HHs) and Tibetan goats (DQs). The core region of the *EPAS1* gene (including all exonic regions) was amplified and sequenced. Subsequently, genomic DNA was extracted from an expanded set of 229 samples to further validate the identified core SNP variation. These included HHs (n = 58), HZs (n = 55), DLs (n = 35), and DQs (n = 81), ensuring a representative sampling across different altitudinal gradients. The amplified and genotyped SNP loci were then analyzed to explore their potential associations with blood physiological phenotypes. The samples used in this part of the experiment were all selected from the aforementioned population.

### 2.2. DNA Extraction from Blood Samples

DNA extraction from blood samples was performed strictly according to the instructions provided in the Blood Genomic DNA Extraction Kit (Tiangen, Beijing, China). Approximately 50 μL of high-concentration DNA solution was extracted from each sample. DNA concentration was measured using a NanoDrop 2000 spectrophotometer (Thermo Fisher Scientific, Waltham, MA, USA), and DNA integrity was assessed by 1% agarose gel electrophoresis to ensure it met the requirements for subsequent gene amplification and sequencing analysis.

### 2.3. Primer Design and PCR Amplification for the EPAS1 Gene

**Figure 2:**
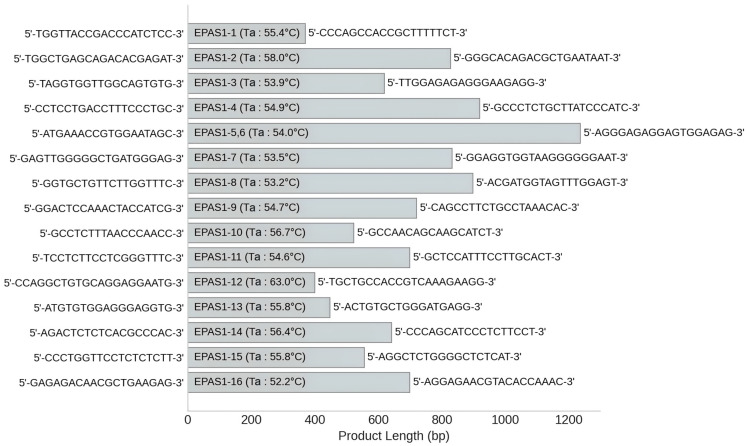
Primer information for the core region of the *EPAS1* gene.

Goat gene sequences for *EPAS1* (Locus: NC_022303.1) were downloaded from the GenBank database of NCBI, and primers for the entire gene region were designed using Premier 5.0 software. All primers were synthesized by TsingKe Biological Technology (Beijing, China); their features are shown in Figure 2.

Initially, 20 individuals (10 from Honghe County [HH] and 10 from Diqing Prefecture [DQ]) were sequenced to identify potential SNPs within the *EPAS1* gene. Based on these results, one SNP was selected for further genotyping in the remaining samples. Genotyping was performed by sequencing, using specific primers (including EPAS1-12) for PCR amplification.

The PCR reactions were carried out in a total volume of 25 μL, consisting of 2 μL genomic DNA, 1 μL of each forward and reverse primer, 12.5 μL of 2 × Taq Master Mix (TsingKe Biological Technology, Beijin, China), and 8.5 μL ddH_2_O. The PCR thermal cycling program used is as follows: initial denaturation at 95 °C for 6 min, followed by 35 cycles, each consisting of denaturation at 94 °C for 50 s, annealing at the temperature specified in Figure 2 for 30 s, and extension at 72 °C for 50 s; the final extension step was performed at 72 °C for 8 min. PCR products were sequenced using the ABI 3730 DNA sequencer. The sequences were edited using Chromas (version 2.6.6) and aligned with MEGA 5.0 software to identify SNPs.

### 2.4. Statistical Analysis

Allele and genotype frequencies were compared using the chi-square test.

To evaluate the physiological and rheological indices of adult goats at different altitudes at the Yunnan–Tibet line and to perform genotype–phenotype association analysis, a two-factor least square analysis without interaction effects was conducted with the following model:

*Y*_*ijk*_ = *μ* + *H*_*i*_/*G*_*i*_ + *S*_*j*_ + *e*_*ijk*_

where *Y_ikj_* is the observed trait value, *µ* is the population mean, *H_i_* is the altitude effect, *G_i_* is the genotype effect, *S_j_* is the sex effect, and *e_ijk_* is the random error, compliant with the *N*(0, $\sigma_{e}^{2}$) distribution.

The GLM (general linear models) procedure in SAS (version 9.0) was used to calculate least squares means, with results expressed as “mean ± SE.” Statistical significance was set at *p* < 0.05 and *p* < 0.01.

### 2.5. 3D Structure Analysis

The functional impact of the Gln 556-Leu (Q556L) polymorphism was predicted using PolyPhen-3 [31]. Five independent predictions were performed for each polymorphism to ensure consistency and reliability. The structure with the highest pTM score was selected for further analysis. Polymorphism site interaction analysis was conducted using PyMOL (Schrödinger, LLC, New York, NY, USA, 2020, version 2.5.6). Hydrogen bonds and electrostatic interactions were examined to assess potential structural and functional alterations. Figures were generated in PyMOL and further annotated and rendered using Adobe Illustrator (Adobe Inc., San Jose, CA, USA, 2021, version 25.1).

## 3. Results

### 3.1. Physiological Adaptation of Yunnan Goats at Different Altitudes

To investigate the high-altitude physiological adaptation phenotypes of Yunnan goats, this study systematically measured blood physiological and rheological parameters of goats distributed across altitudes ranging from 500 m to 3500 m in Yunnan Province (Table 1). The results indicated significant changes in blood parameters with increasing altitude as atmospheric oxygen levels decreased (*p* < 0.01).

RBC and HGB increased significantly with altitude, from 11.97 ± 2.12 × 10^12^/L and 72.10 ± 12.49 g/L in the local goats at 500 m (HHs) to 17.40 ± 2.25 × 10^12^/L and 101.01 ± 9.47 g/L in the DQs at 3500 m (*p* < 0.01). HCT followed a similar trend, rising from 19.58 ± 3.23% to 30.02 ± 3.11% (*p* < 0.01). PV peaked at 2.24 ± 0.45 mPa·s in the DLs at 2500 m (*p* < 0.01) but decreased to 1.64 ± 0.24 mPa·s in the DQs at 3500 m. FB and EI also showed significant changes, with fibrinogen at 4.92 ± 1.00 g/L in the DLs at 2500 m, significantly higher than 3.66 ± 0.39 g/L in the DQs at 3500 m (*p* < 0.01). Furthermore, with increasing altitude, both EI and LSFR declined, while YS peaked at 4.97 ± 2.55 mPa in the DLs at 2500 m (*p* < 0.01).

### 3.2. Identification of EPAS1 Gene SNPs and Genotyping Results

**Figure 3:**
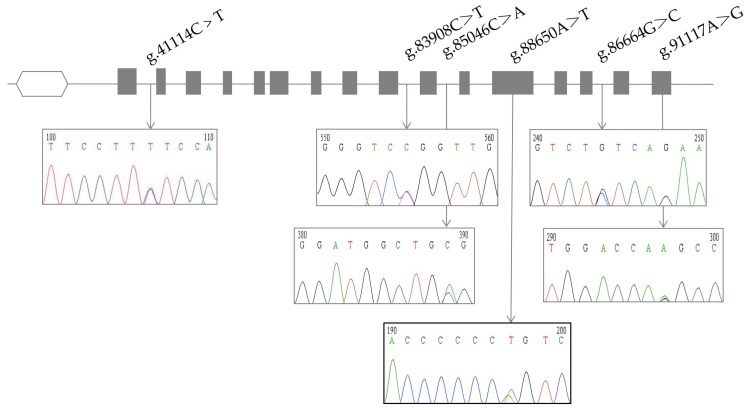
SNP variant information of the *EPAS1* gene.

The *EPAS1* gene is a critical regulatory gene for hypoxia adaptation in high-altitude mammals. In this study, Sanger sequencing was used to analyze the core regions of the *EPAS1* gene in high-altitude DQs and low-altitude HHs. A total of six SNPs were identified (Figure 3). Among them, *g.86650 A>T* (Q556L) is a missense mutation that results in an amino acid substitution from glutamine (Q) to leucine (L) at position 556, suggesting a potential functional impact on protein structure or activity. Significant differences in genotype and allele frequencies at this position were observed between the two populations (*p* < 0.01). In the DQs (high-altitude, 3500 m), the *T* allele frequency was 0.85, with the *TT* genotype accounting for 0.70. In contrast, in the HHs (low-altitude, 500 m), the *A* allele frequency was 0.70, and the *AA* genotype accounted for 0.60. Similarly, for *g.41114C>T*, genotype distributions differed significantly between high- and low-altitude populations (*p* < 0.01), with the *TT* genotype frequency being 0.70 in DQs but only 0.20 in HHs, suggesting a potential role in hypoxia adaptation. *g.91117A>G* showed significant differentiation between high- and low-altitude populations (*p* < 0.01), with the *GG* genotype at 0.57 in DQs and fixed (1.00) in HHs. However, as a synonymous SNP with no amino acid change, its functional relevance remains unclear, and it was not included in the association analysis. In contrast, the remaining four SNPs (*g.83908 C>T*, *g.85046 C>A*, *g.88664 G>C*, and *g.91117 A>G*) did not show significant differences in genotype or allele frequencies between high- and low-altitude populations (*p* > 0.05).

To further validate the role of *g.86650 A>T* in altitude adaptation, we expanded genotyping analysis to include goat populations across different altitudes in Yunnan Province (DQ: 3500 m, DL: 2500 m, HZ: 1500 m, and HH: 500 m). The results showed that the *T* allele frequency increased with altitude. In the DQs, the *T* allele frequency was 0.78, with the *TT* genotype being dominant (0.62), whereas in the HHs, the *T* allele frequency decreased to 0.41, with a value of 0.36 for the *AA* genotype (*p* < 0.01) (Table 2).

### 3.3. Impact of Gln 556-Leu (Q556L) Polymorphism on Hematological and Hemorheological Traits Across Altitudes

To evaluate the effects of the Q556L missense polymorphism on hematological and hemorheological indices, a genotype–phenotype association analysis was conducted in goat populations across different altitudes in Yunnan Province (Table 3 and Table 4). In the DQ (3500 m), HZ (1500 m), and HH (500 m) populations, no significant associations were observed between genotypes and either hematological or hemorheological indices (*p* > 0.05). However, in the DL (2500 m) population, individuals with the *TT* genotype (plateau type) exhibited significantly higher red blood cell (RBC) and hemoglobin (HGB) concentrations compared to those with the *AA* genotype (lowland type) (RBC: 16.02 ± 2.57 vs. 12.37 ± 1.88 × 10^12^/L, HGB: 101.00 ± 16.09 vs. 79.83 ± 12.99 g/L, *p* < 0.05).

In addition, in the DL population, individuals with the *TT* genotype had significantly higher hematocrit (HCT) values compared to those with the *AA* genotype (29.08 ± 4.19% vs. 23.54 ± 3.73%, *p* < 0.05), accompanied by elevated blood viscosity indices such as whole-blood low-shear relative index (WLS). However, *TT* genotype individuals exhibited significantly lower red blood cell aggregation (EI and EC) compared to the *AA* genotype (*p* < 0.05), suggesting that the reduced aggregation capacity of red blood cells may effectively mitigate the adverse effects of blood flow resistance.

### 3.4. Structural Analysis of the Gln 556-Leu (Q556L) Polymorphism

**Figure 4:**
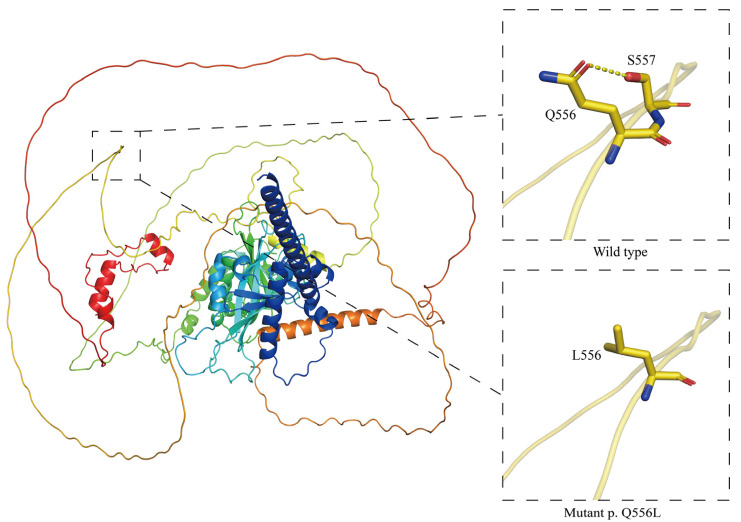
Structural comparison of wild-type and Q556L mutant proteins, highlighting hydrogen bond disruption and local conformational changes.

The Gln 556-Leu (Q556L) polymorphism’s functional impact was assessed using structural modeling and interaction analysis Figure 4. The comparison between the wild-type and mutant forms revealed significant alterations in local structural stability and hydrogen bonding.

In the wild-type protein, Q556 (glutamine) formed a stable hydrogen bond with S557 (serine), stabilizing the local structure. In the mutant, the substitution of hydrophobic leucine disrupted this bond, reducing stability and altering the region’s surface properties, potentially affecting biomolecular interactions.

Visualization using PyMOL (version 2.5.6) highlighted these changes, with PolyPhen-3 predicting a deleterious impact on functionality. The polymorphism’s structural effects suggest impaired adaptation to hypoxic conditions, emphasizing its potential role in protein dysfunction. The figures generated in this study illustrate the structural impact of the polymorphism, offering deeper insights into its biological implications.

## 4. Discussion

High-altitude hypoxia poses significant challenges to the survival of flora, fauna, and humans. Populations indigenous to high-altitude regions exhibit distinct adaptive characteristics. For example, Tibetans and Sherpas maintain relatively low hemoglobin concentrations [32], which significantly reduces the risk of polycythemia [33,34] and chronic mountain sickness [35] compared to lowland populations. Similarly, this study found that high-altitude Tibetan goats significantly enhance oxygen transport capacity by increasing red blood cell count (RBC), hemoglobin concentration (HGB), and hematocrit level (HCT). This adaptive strategy is consistent with that observed in other high-altitude mammals, such as Tibetan donkeys [36], Tibetan pigs [37], and Tibetan horses [38]. These findings provide further evidence supporting the existence of convergent mechanisms of high-altitude hypoxia adaptation across species.

While increased hemoglobin concentration is an effective compensatory mechanism in hypoxia, excessive erythrocytosis may elevate blood viscosity, impairing tissue perfusion and oxygen delivery [37,39]. This study revealed a unique mechanism for regulating blood rheology in Tibetan goats. Despite slightly higher plasma viscosity and erythrocyte aggregation compared to low-altitude goats, their whole-blood high-shear relative index (WHS) and Casson viscosity (CV) were significantly lower, indicating maintained erythrocyte deformability. This trait, also observed in high-altitude species like yaks and Tibetan sheep, mitigates the adverse effects of increased blood viscosity on microcirculation by optimizing erythrocyte deformability.

At the molecular level, hypoxia-sensing gene pathways play central roles in regulating erythropoiesis, angiogenesis, cardiopulmonary adjustment, and energy metabolism. *EPAS1*, encoding HIF-2α, is a key member of the hypoxia signaling pathway. By regulating downstream genes such as erythropoietin (*EPO*) and vascular endothelial growth factor (*VEGF*), *EPAS1* enhances hypoxic adaptation [40,41]. Under normoxia, HIF-2α is hydroxylated by PHD and degraded via VHL-mediated ubiquitination. However, under hypoxia, HIF-2α translocates to the nucleus, dimerizes with HIF-1β, and activates downstream transcription [40]. In humans, SNP variations in *EPAS1* are strongly associated with the lower hemoglobin levels observed in Tibetans [42,43,44]. Similarly, specific variations in *EPAS1* are significantly correlated with hemoglobin concentration in yaks [45], indicating the conserved role of this gene in high-altitude adaptation.

The structural analysis further demonstrated the potential impact of the missense polymorphism (*g.86650 A>T*, p.Gln556Leu) in exon 12 of *EPAS1*. Protein modeling revealed that Q556 (glutamine) in the wild-type protein formed a stable hydrogen bond with S557 (serine), maintaining local structural stability. In contrast, the substitution to L556 (leucine) disrupted this hydrogen bond due to the change from a polar to a hydrophobic side chain, reducing structural stability. Such alterations may impair HIF-2α functionality, affecting downstream gene regulation and reducing hypoxic adaptation.

The sequencing results indicate a significant increase in the frequency of the T allele at this position with elevation (*p* < 0.05), with the high-altitude genotype (*TT*) predominant in Tibetan goats. Association analysis showed that in the DL (2500 m) population, individuals with the *TT* genotype exhibited significantly higher RBC, HGB, and HCT levels than those with the *AA* genotype (*p* < 0.05), suggesting that this polymorphism enhances oxygen transport capacity to promote high-altitude adaptation. Although the *TT* genotype was associated with increased blood viscosity (e.g., WLS), it also showed significantly reduced erythrocyte aggregation (EI and EC) (*p* < 0.05), likely mitigating the adverse effects of increased viscosity on blood circulation by maintaining erythrocyte deformability.

Notably, no significant differences in hematological or hemorheological traits were observed between the *TT* and *AA* genotypes in the DQ (3500 m), HZ (1500 m), and HH (500 m) populations. This may be attributed to the smaller sample size of the *AA* genotype in low-altitude populations, limiting statistical power. Additionally, Song et al. [29] reported a significant association between the p.Gln556Leu polymorphism and mean corpuscular hemoglobin concentration, a result not replicated in this study, suggesting that the functional effects of *EPAS1* may vary with genetic background and environmental factors.

In conclusion, this study further validates the critical role of *EPAS1* in high-altitude adaptation and provides new insights into the molecular mechanisms underlying adaptive evolution in high-altitude animals. Future studies with larger sample sizes and functional validation experiments are warranted to investigate the adaptive effects and regulatory mechanisms of this gene under varying environmental conditions.

## 5. Conclusions

This study highlights the critical role of the *EPAS1 g.86650 A>T* (p.Gln556Leu) polymorphism in enhancing oxygen transport capacity in Tibetan goats under hypoxic conditions. The polymorphism was associated with increased RBC, HGB, and HCT levels, particularly in the DL population, indicating its significance in high-altitude adaptation. Despite elevated blood viscosity, reduced erythrocyte aggregation indices (EI and EC) in the *TT* genotype suggest a compensatory mechanism to maintain microcirculation.

The structural analysis showed that the Q556L substitution disrupted hydrogen bonding, potentially impairing *EPAS1* functionality. These findings provide new insights into hypoxia adaptation mechanisms and offer a foundation for improving livestock breeding strategies in high-altitude regions.

## Figures and Tables

**Table 1:** Hematological and hemorheological indicators of goats at different altitude gradients.

	DQ (n = 81)	DL (n = 35)	HZ (n = 55)	HH (n = 58)
	3500 m	2500 m	1500 m	500 m
Red Blood Cell (RBC, 10¹²/L)	17.40 ± 2.25 ^A^	15.23 ± 2.34 ^B^	13.98 ± 2.48 ^C^	11.97 ± 2.12 ^D^
Hemoglobin Concentration (HGB, g/L)	101.01 ± 9.47 ^A^	93.18 ± 14.90 ^B^	76.87 ± 13.38 ^C^	72.10 ± 12.49 ^D^
Hematocrit (HCT, %)	30.02 ± 3.11 ^A^	27.33 ± 3.76 ^B^	25.14 ± 4.19 ^C^	19.58 ± 3.23 ^D^
Mean Corpuscular Volume (MCV, fL)	17.34 ± 1.85 ^C^	18.14 ± 1.71 ^A^	18.07 ± 1.79 ^AB^	16.50 ± 1.51 ^D^
Mean Corpuscular Hemoglobin (MCH, pg)	5.77 ± 0.54 ^C^	6.13 ± 0.46 ^A^	5.44 ± 0.41 ^D^	5.99 ± 0.43 ^B^
Mean Corpuscular Hemoglobin Concentration (MCHC, g/L)	335.44 ± 17.80 ^B^	340.85 ± 22.78 ^B^	305.00 ± 14.62 ^C^	366.63 ± 12.51 ^A^
Red Cell Distribution Width (RDW, %)	20.57 ± 1.84 ^A^	20.42 ± 1.58 ^A^	20.23 ± 1.71 ^A^	20.04 ± 1.28 ^A^
Plasma Viscosity (PV, mPa·s)	1.64 ± 0.24 ^C^	2.24 ± 0.45 ^A^	1.83 ± 0.30 ^B^	1.52 ± 0.29 ^C^
Fibrinogen (FB, g/L)	3.66 ± 0.39 ^C^	4.92 ± 1.00 ^A^	4.03 ± 0.65 ^B^	3.34 ± 0.63 ^D^
Whole-Blood Low-Shear Relative Index (WLS)	6.88 ± 1.21 ^A^	6.07 ± 1.59 ^B^	5.78 ± 1.25 ^B^	6.59 ± 1.58 ^A^
Whole-Blood Medium-Shear Relative Index (WMS)	1.83 ± 0.24 ^B^	1.61 ± 0.43 ^C^	1.69 ± 0.33 ^C^	2.14 ± 0.43 ^A^
Whole-Blood High-Shear Relative Index (WHS)	1.39 ± 0.19 ^B^	1.23 ± 0.38 ^C^	1.32 ± 0.28 ^BC^	1.70 ± 0.31 ^A^
Erythrocyte Aggregation Index (EI)	4.97 ± 0.69 ^B^	5.36 ± 2.07 ^A^	4.42 ± 0.78 ^C^	3.83 ± 0.42 ^D^
Erythrocyte Aggregation Coefficient (EC)	3.41 ± 0.47 ^B^	3.67 ± 1.42 ^A^	3.04 ± 0.54 ^C^	2.63 ± 0.29 ^D^
Casson Viscosity (CV, mPa·s)	1.88 ± 0.17 ^B^	2.19 ± 0.67 ^A^	2.02 ± 0.43 ^A^	2.20 ± 0.34 ^A^
Erythrocyte Intracellular Viscosity (RBCIV, mPa·s)	0.67 ± 0.07 ^A^	0.90 ± 0.18 ^A^	0.79 ± 0.12 ^A^	0.61 ± 0.11 ^A^
Low-Shear Flow Resistance (LSFR, 10⁹ SI)	33.85 ± 4.95 ^B^	39.83 ± 10.71 ^A^	30.98 ± 5.09 ^C^	28.73 ± 4.17 ^C^
Medium-Shear Flow Resistance (MSFR, 10⁹ SI)	21.16 ± 1.92 ^B^	24.37 ± 5.15 ^A^	21.34 ± 3.62 ^B^	22.23 ± 3.37 ^B^
High-Shear Flow Resistance (HSFR, 10⁹ SI)	16.00 ± 1.33 ^B^	18.52 ± 4.79 ^A^	16.65 ± 3.06 ^B^	18.03 ± 2.97 ^AB^
Yield Stress (YS, mPa)	3.99 ± 0.99 ^B^	4.97 ± 2.55 ^A^	3.31 ± 0.91 ^C^	2.64 ± 0.54 ^D^

The data in the same line were compared; means with different capital letters are significantly different (*p* < 0.01).

**Table 2:** Genotype and allele frequencies of the *g.86650 A>T* position in goat populations across different altitudes.

Population	Genotype Frequencies			Allele Frequencies		*p* Value
	AA	AT	TT	A	T	
DQ	0.06	0.32	0.62	0.20	0.80	<0.01
DL	0.51	0.31	0.17	0.70	0.30	
HZ	0.47	0.27	0.25	0.61	0.39	
HH	0.40	0.40	0.20	0.60	0.40	

**Table 3:** Association analysis of hematological traits with genotypes.

Group	Genotype	RBC (10^12^/L)	HGB (g/L)	HCT (%)	MCV (fL)	MCH (pg)	MCHC (g/L)	RDW (%)
DQ	*TT* (50)	18.81 ± 3.06	113.14 ± 18.19	31.72 ± 5.15	16.91 ± 1.17	5.95 ± 0.33	355.90 ± 13.63	20.53 ± 1.52
	*AT* (26)	19.16 ± 1.30	113.77 ± 6.43	31.89 ± 2.23	16.71 ± 1.09	5.90 ± 0.29	356.65 ± 11.11	20.97 ± 1.33
	*AA* (5)	18.92 ± 2.36	112.60 ± 8.88	31.56 ± 2.06	16.84 ± 1.55	5.94 ± 0.38	356.00 ± 11.66	20.02 ± 1.21
DL	*TT* (6)	16.02 ± 2.57 ^a^	101.00 ± 16.09 ^a^	29.08 ± 4.19 ^a^	18.22 ± 0.39	6.25 ± 0.08	346.17 ± 8.75	20.62 ± 1.40
	*AT* (11)	14.50 ± 2.12 ^a^	90.91 ± 14.24 ^ab^	26.29 ± 3.90 ^ab^	18.21 ± 1.21	6.22 ± 0.29	345.00 ± 9.61	19.70 ± 1.42
	*AA* (18)	12.37 ± 1.88 ^b^	79.83 ± 12.99 ^b^	23.54 ± 3.73 ^b^	19.11 ± 1.29	6.41 ± 0.35	338.61 ± 12.23	19.46 ± 1.43
HZ	*TT* (14)	12.88 ± 2.46	69.21 ± 15.11	22.77 ± 4.67	17.76 ± 1.54	5.31 ± 0.47	303.07 ± 12.20	21.52 ± 1.47
	*AT* (15)	12.79 ± 3.04	69.47 ± 18.99	22.96 ± 5.72	18.02 ± 1.40	5.35 ± 0.39	300.13 ± 12.82	21.66 ± 1.29
	*AA* (26)	13.39 ± 2.31	73.04 ± 13.07	24.24 ± 4.17	18.27 ± 2.11	5.41 ± 0.37	300.92 ± 16.03	22.27 ± 1.84
HH	*TT* (11)	14.95 ± 2.44	94.55 ± 10.74	26.89 ± 2.73	18.34 ± 2.67	6.34 ± 0.61	351.18 ± 18.36	19.95 ± 1.81
	*AT* (26)	14.88 ± 2.24	93.69 ± 12.77	26.93 ± 3.28	18.39 ± 2.61	6.31 ± 0.68	348.12 ± 29.90	21.58 ± 2.06
	*AA* (21)	14.35 ± 2.79	95.38 ± 14.24	25.97 ± 5.75	18.07 ± 2.10	6.74 ± 1.18	352. 58 ± 13.75	21.60 ± 3.49

Values in the same row with different lowercase letters indicate significant differences *(p* < 0.05), while values without letters are not significantly different (*p* > 0.05).

**Table 4:** Association analysis of hemorheological traits with genotypes.

Group	Genotype	PV (mPa·s)	FB (g/L)	WLS	WMS	WHS	EI	EC	CV (mPa·s)	RBCIV (mPa·s)	LSFR (10^9^ SI)	MSFR (10^9^ SI)	HSFR (10^9^ SI)	YS (mPa)
DQ	*TT* (50)	1.16 ± 0.75	2.55 ± 1.64	5.73 ± 3.91	1.73 ± 1.18	1.36 ± 0.93	2.95 ± 1.96	2.02 ± 1.35	1.84 ± 1.24	0.45 ± 0.30	27.23 ± 18.18	19.28 ± 12.92	15.19 ± 10.20	2.77 ± 1,86
	*AT* (26)	1.28 ± 0.73	2.83 ± 1.61	5.85 ± 3.35	1.78 ± 1.02	1.41 ± 0.80	3.20 ± 1.80	2.20 ± 1.24	1.98 ± 1.12	0.51 ± 0.29	28.89 ± 16.22	20.61 ± 11.61	16.27 ± 9.19	2.91 ± 1.64
	*AA* (5)	1.40 ± 0.80	3.08 ± 1.77	5.94 ± 3.44	1.84 ± 1.08	1.46 ± 0.86	3.26 ± 1.84	2.24 ± 1.26	2.15 ± 1.22	0.56 ± 0.32	30.66 ± 17.17	22.25 ± 12.51	17.65 ± 9.95	3.02 ± 1.71
DL	*TT* (6)	2.35 ± 0.34	5.16 ± 0.76 ^a^	8.64 ± 1.90 ^a^	1.82 ± 0.50 ^a^	1.28 ± 0.39	6.93 ± 0.92 ^b^	4.75 ± 0.63 ^b^	2.34 ± 0.86 ^a^	0.94 ± 0.14	60.21 ± 14.87 ^a^	29.91 ± 9.49 ^a^	21.08 ± 7.26 ^a^	8.81 ± 1.77 ^ab^
	*AT* (11)	2.51 ± 0.45	5.53 ± 1.00 ^a^	8.06 ± 1.97 ^ab^	1.57 ± 0.54 ^ab^	1.08 ± 0.43	8.13 ± 2.28 ^a^	5.58 ± 1.57 ^a^	1.99 ± 0.81 ^ab^	1.01 ± 0.18	59.05 ± 11.04 ^a^	26.80 ± 7.94 ^ab^	18.34 ± 6.53 ^ab^	9.39 ± 2.13 ^a^
	*AA* (18)	2.46 ± 0.29	3.76 ± 1.54 ^b^	6.55 ± 1.76 ^b^	1.24 ± 0.39 ^b^	1.14 ± 0.30	8.14 ± 2.00 ^a^	5.59 ± 1.37 ^a^	1.53 ± 0.59 ^b^	0.98 ± 0.12	47.44 ± 11.30 ^b^	21.07 ± 6.32 ^b^	14.29 ± 4.88 ^b^	7.64 ± 2.05 ^b^
HZ	*TT* (14)	1.84 ± 0.71	4.05 ± 155	6.05 ± 1.74	1.81 ± 0.54	1.42 ± 0.44	4.32 ± 0.56	3.96 ± 0.38	2.02 ± 0.41	0.74 ± 0.28	30.47 ± 5.09	21.28 ± 3.67	16.71 ± 3.12	3.17 ± 0.75
	*AT* (15)	1.83 ± 0.20	4.04 ± 0.44	5.82 ± 0.99	1.72 ± 0.33	1.35 ± 0.28	4.36 ± 0.65	3.00 ± 0.45	2.08 ± 0.49	0.73 ± 0.08	31.56 ± 4.26	21.97 ± 4.15	17.24 ± 3.68	3.30 ± 0.60
	*AA* (26)	1.76 ± 0.48	3.86 ± 1.06	5.97 ± 1.62	1.74 ± 0.44	1.36 ± 0.35	4.42 ± 0.64	3.03 ± 0.44	2.12 ± 0.30	0.70 ± 0.19	33.11 ± 6.04	22.58 ± 3.07	17.61 ± 2.43	3.53 ± 1.03
HH	*TT* (11)	1.39 ± 0.33	3.05 ± 0.73	8.68 ± 2.00	2.13 ± 0.45	1.58 ± 0.33	5.49 ± 0.46	3.77 ± 0.32	1.70 ± 0.15	0.55 ± 0.13	34.59 ± 5.03	19.87 ± 2.01	14.71 ± 1.37	4.42 ± 0.90
	*AT* (26)	1.36 ± 0.39	2.98 ± 0.85	8.81 ± 1.72	2.10 ± 0.37	1.54 ± 0.28	5.74 ± 0.71	3.94 ± 0.49	1.63 ± 0.38	0.54 ± 0.16	34.47 ± 6.19	19.35 ± 3.90	14.22 ± 3.10	4.52 ± 0.92
	*AA* (21)	1.26 ± 0.23	2.77 ± 0.50	10.12 ± 3.61	2.45 ± 0.90	1.80 ± 0.68	5.66 ± 0.61	3.88 ± 0.42	1.72 ± 0.30	0.50 ± 0.09	36.01 ± 6.29	20.33 ± 3.36	14.97 ± 2.55	4.69 ± 1.00

Values in the same row with different lowercase letters indicate significant differences (*p* < 0.05), while values without letters are not significantly different (*p* > 0.05).

## Data Availability

The data presented in this study are available upon request from the corresponding author.
